# Correlates of Climate Change Action Communication Modalities in the United States

**DOI:** 10.3390/cli11060125

**Published:** 2023-06-07

**Authors:** Carl Latkin, Lauren Dayton, Haley Bonneau, Kennedy Countess, Zoé Hendrickson, Carol Vidal

**Affiliations:** 1 Department of Health, Behavior and Society, Bloomberg School of Public Health, Johns Hopkins University, Baltimore, MD 21205, USA;; 2 Department of Psychiatry, School of Medicine, Johns Hopkins University, Baltimore, MD 21205, USA;

**Keywords:** climate change, activism, collective action, barriers, communication

## Abstract

Communicating about actions to address climate change is critical to mobilize collective actions, and enact policies for climate change mitigation (prevention) and adaptation to climate change. The current study assessed factors associated with climate change action (CCA) communications in the US. Respondents were recruited through Prolific, an online survey research platform. The sample was restricted to the 599 respondents who reported that the issue of climate change was extremely or very important to them. Key outcome variables included (1) talking to family/friends about CCA, (2) texting/emailing family/friends about CCA, and (3) posting or sharing a post on social media about CCA. Multinomial logistic regression models examined correlates of CCA communications. Descriptive and injunctive social norms, barriers to CCA, and climate change distress were consistently significantly associated with engaging in the three CCA communication modalities in the prior month compared to never. This study’s results suggest that talking with peers is the most common form of CCA communication, and is associated with social norms and distinct barriers to CCA. Organizations that address climate change should consider utilizing dialogical approaches to shift social norms related to CCA, and foster CCA communications and address barriers to CCA.

## 1. Introduction

Massive infrastructure and other societal changes are necessary to mitigate the effects of and adapt to climate change. Despite a large body of research on the adverse impacts of climate change on all aspects of human and planetary health, political action to prevent or mitigate climate change has been stymied by opposition from the fossil fuel industry, corporate interests, as well as political and ideological opposition to addressing the climate [1,2]. Overcoming opposition to climate change mitigation policies requires a shift in the public opinion landscape that supports the enactment of major climate mitigation policies for reducing fossil fuel consumption and increasing renewable energy sources. Climate change action (CCA) communication is one strategy that may help create a public opinion landscape supportive of climate change mitigation policies [3,4]. CCA communication refers to communication about climate change actions, such as voting and supporting candidates who support climate change mitigation policies, lobbying policymakers to enact meaningful legislation, donating to organizations that address climate change, and attending protests or rallies.

CCA communication can occur through one-on-one communication or through communication on social media platforms [5]. Initially, research on climate change communication focused on how to accurately present the science that validated the existence and impact of climate change on individual-level behaviors. For example, a study in Northern Canada found that talking with family and friends about climate change was associated with a measure of individual-level climate change behaviors, such as recycling and reducing beef consumption [6]. However, to increase the probability that climate change communication will lead to actions, communication about climate change needs to provide information about the actions necessary to address it, as well as encourage, support, and symbolically reward activism [7,8]. The current study examined factors associated with engaging in communication about climate change actions (i.e., CCA communication).

CCA communication can differ from dialogical to transmissive. Carey contrasts a more dialogical, ritual view of communication—which sees communication as a horizontal process rooted in sharing and community that is central to establishing and reinforcing shared societal understandings, meanings, and values—with transmissive communication, where the dissemination of information is the main goal of this more vertical communicative act [9]. There are multiple strategies by which CCA communication can lead to changes in beliefs and behaviors. From a transmissive view of communication, one mechanism is diffusing information about CCA through social networks. Social network and diffusion of innovations theories posit that information and behaviors are spread through social networks [10]. At the same time, CCA communication can also lead to changes in beliefs and behaviors through social influence mechanisms, including modeling and vicarious learning, social comparison process, cues to action, and social rewards [11]. From a more ritual view of communication, communication between peers can also heighten one’s social identity or sense of who they are based on their social network, and establish and reinforce shared norms and values related to CCA [8,12,13]. Research on climate change activism suggests that a heightened social identity can lead to a greater probability of engaging in collective actions that are consistent with the social identity [13,14].

The ritual view of communication emphasizes that CCA communication may change attitudes and promote collective climate action through the changing and reinforcing of social norms. Social norms may in turn promote the acceptability of CCA communication. Communications provide the recipient with information on the views and opinions of other important people [15]. Talking about a behavior or voicing an opinion can lead to increased saliency, and enhance the view that the behavior or opinion is frequently endorsed and viewed as normative. For example, if an individual perceives that others in their social network are engaged in climate change action, they might perceive climate change action as normative. There is a large body of literature documenting the impact of norms on environmental behaviors [5,15–18]. For example, one social norm intervention that has been found to reduce home energy use informs individuals that their energy use of gas and electricity is high, lower, or similar to their neighbors. Those individuals who are given information that their use is high tend to reduce their energy use [15]. Some common categorizations of social norms are injunctive and descriptive norms. Injunctive norms are evaluative and are the perception of whether peers believe one should or should not engage in a behavior. Descriptive norms are perceptions of the degree to which peers engage in the behavior.

Taken together, social network, communication, and behavior change theories suggest that CCA communication can diffuse information about climate change activism through social networks, and establish and reinforce peers’ beliefs and behaviors related to climate change mitigation. In the current study, we examined if injunctive or descriptive social norms related to climate change action were associated with CCA communication. It was hypothesized that both norms would be associated with CCA communication.

Intrapersonal factors also influence behaviors. Negative emotions have been linked to climate change action. One experimental study found that experiencing negative emotions related to climate change increased the willingness to take action on climate change [19]. A recent study found that individuals who reported high levels of climate change psychological distress were more likely to engage in climate change actions such as voting and supporting environmental organizations [20]. However, this study did not examine if climate change psychological distress is linked to face-to-face and online climate change action communications. As communications with family and friends may take less effort and time than other climate change actions, such as volunteering, it is possible that different factors may influence the frequency and types of CCA communications than influence climate change actions; hence, we examined if higher levels of climate change psychological distress were associated with CCA communications.

Communication about climate change has also been affected by the “spiral of silence” [21,22]. The “spiral of silence” hypothesis suggests that if people do not talk about a topic, due in part to the perceived inappropriateness of such discussions, this can lead to misconceptions about others’ attitudes, as well as discrepancies between individual perceptions and relevant injunctive and descriptive social norms. The spiral can also be formed due to concern about offending others who hold opposing views. In the US, climate change is highly politicized, and individuals may refrain from talking about climate change with peers affiliated with different political parties. However, there is evidence that members of the Republican political party in the US, which has historically opposed climate protection policies and favors private interests, are more concerned about climate change than perceived to be by the public [23]. The spiral of silence between peers from different political parties, therefore, may be due to perceived, rather than actual, differences in beliefs. The promotion of CCA communication can normalize discussion about climate change and help overcome the spiral of silence that currently affects CCA communication.

Barriers to engaging in climate change actions may also be associated with CCA communications. Given that the majority of US residents are concerned about climate change, but few engage in collective actions, the gap between concern and action may be influenced by barriers to action. Previously documented barriers to collective action included time, competing priorities, lack of resources, self-efficacy, information on effective actions, and skills to engage in these actions [24–27]. However, there is little research on how these potential barriers to CCA communication intersect. For example, do barriers correlate, and are there underlying factors that influence them? Information on the structure of CCA communication barriers may be helpful for developing programs to address them. For example, if barriers are highly correlated, it may suggest that respondents are identifying post hoc reasons for not engaging in CCA communication, or that perhaps there are larger cross-cutting or latent factors that influence these barriers for CCA communication. Whereas, if reported barriers are independent, it may suggest that there are unique barriers that might be addressed. In the current study, we first conducted a factor analysis to examine barriers to CCA communication, and then examined how these factors may impede CCA communications.

CCA communication can occur using different modalities that have unique diffusion properties, as well as facilitators and barriers. For example, face-to-face may be more influential than other communication modalities based on the potential for dialogical rather than transmissive communication; however, electronic mediated communications on social media, email, and texting have the advantage of diffusing information to a large audience that may be socially or geographically distant. Social media can influence public opinion and be used as a platform for climate change activism [28–30]. There is a substantial body of research on social media and climate change communications [31]. One study assessed two years of data from a Facebook fan-based page entitled “Global Climate Change Awareness” [32]. The investigators found that the frequency of posts made to the page did not significantly promote user engagement, but did heighten the visibility of the content. Most of the social media research on climate change has focused on Twitter. Content and social network analyses have examined social media postings on climate change and content analyses of the posts [33]. Tweets have also been examined to determine factors that may lead to retweets, and climate change-related social media posts have been analyzed for content and diffusion [34]. Tyangi et al. reported that deniers of climate change on Twitter were more hostile towards people who believe in the human causes of climate change compared to those who do not [35]. Other studies have examined the social network structure of climate change tweets, but little is known about who posts materials on climate change action, and if those who post also tend to talk to others about climate change actions [36,37]. The current study design allowed us to examine whether modes of CCA communications are correlated, and whether the same factors predict CCA communications across different communication modalities, including talking, posting on social media, and email/texting about climate change actions.

Together, we examined whether descriptive and injunctive social norms, climate change distress, and self-reported barriers to climate change actions were associated with CCA communications. Given the unique nature of climate change of having a greater negative impact on future generations, we also examined whether perceptions of youth climate change actions were also associated with CCA communications. Understanding factors associated with CCA communications may help develop more effective methods to increase CCA communications and influence climate change action.

## 2. Methods

### 2.1. Measures

Participants were recruited on the online research platform Prolific, which utilizes rigorous screening methods and has been found to outperform other online research panels and platforms [38]. Participants were recruited for an RCT of an intervention to promote network members’ communications about either climate change or the COVID-19 vaccine. Individuals were required to be aged 18 or older and reside in the US. To ensure that we had a racially diverse sample and could examine CCA communications among minority groups that will be disproportionally impacted by climate change, we oversampled Black and Hispanic participants. Attention and validity checks were included in the survey. Checks included survey questions with extremely low probabilities for affirmative responses, such as deep-sea fishing in Alaska and having multiple appendages removed. We also repeated certain questions to assess consistency. Finally, we assessed the completion time and verified survey completeness. Participants who did not pass all of the validity checks were excluded from the analyses. Participants provided informed consent, and study protocols were approved by the Bloomberg School of Public Health IRB.

As assessing CCA communications behaviors is most relevant to those individuals who are concerned about climate change, we used a screening item to identify individuals based [39] on the survey item “How important is the issue of climate change to you personally?” The response categories were “Extremely important”, “Very important”, “Somewhat important”, “Not too important”, and “Not at all important”. We restricted the sample to those who reported that climate change was personally “Extremely important” or “Very important” to them.

As there are different conceptions of climate change actions for the present study, participants were primed as to the meaning of climate change action by a series of questions asking if they had engaged in six types of climate change actions [39]. These included (1) personally wrote letters, emailed, or phoned government officials to urge them to take action to reduce climate change; (2) voted for candidates who support measures to reduce climate change; (3) signed an online petition or provided their name and email address to an environmental organization to send an email to a policy maker urging action on climate change; (4) volunteered with organizations working to curb climate change; (5) donated money to organizations working to reduce climate change; and (6) attended protests or rallies to reduce climate change. Responses were binary (yes/no).

The key CCA communications outcomes were (1) when did you last talk to your family/friends about climate change action, (2) how often have you texted/emailed your friends/family about climate change action, and (3) on social media, when did you last do the following actions: posted or shared a post about the need for action on climate change. Response options for the three CCA communication questions were “Never”, “More than a year ago”, “Past year”, and “Past month”. To analyze recency of CCA communication, a three-level outcome variable was created to compare never, more than a month ago, and past month CCA communication. Participants were also asked, “How often have you posted on social media about climate change actions?” Response options were “Never”, “Less than monthly”, “Less than weekly”, “Weekly”, and “Daily”.

Two items assessed social norms. One assessed descriptive norms: “Of your friends/family, how many do you think are involved in climate change actions?” The other assessed injunctive norms: “How many friends/family would encourage you to take action to reduce climate change?” The response categories were “None (0%), Some (25%), Half (50%), Most (75%), All (100%)”.

The 5-item brief climate change distress scale assessed psychological climate change distress symptoms of anxiety, depression, worry, and feelings of helplessness and hopelessness associated with thinking about climate change; e.g., “News about climate change tends to depress me” and “Thinking about climate change makes me anxious” [20]. The scale’s Cronbach alpha was 0.83. As a balance to the negative emotions of psychological distress, one item assessed inspiration to engage in climate change action: “When you see younger adults urging action on climate change, how does that impact your personal interest in addressing climate change?” The response options were “More interested”, “Less interested”, and “Makes no difference”. To assess distress due to peers reacting negatively to the respondent engaging in climate change action, the following survey item was included: “I would feel very uncomfortable telling my friends/family if I was involved in actions to combat climate change. Such changes may include: supporting and voting for candidates who prioritize legislation to curb climate change/letter writing, emailing and calling legislators to urge them to take action on climate change/volunteering with or donating to organizations working to curb climate change”. The response options were “Strongly agree”, “Agree”, “Neither agree or Disagree”, “Disagree”, or “Strongly disagree”.

Based on prior research, a set of barriers to climate action was included [40]. Respondents were asked to indicate reasons for their lack of involvement in climate change activism via the question “Please rate the reasons you haven’t been involved in climate change advocacy”: (1) “Too busy”, (2) “Don’t know how to get involved”, (3) “Other people are better at it than me”, (4) “If I do get involved, organizations will ask me for money”, (5) “Haven’t been asked”, (6) “Activities like letter writing aren’t appealing”, (7) “I haven’t been trained”, (8) “Not encouraged to become involved”, (9) “Not something I think about a lot”, (10) “What I could do will not have an impact”, (11) “Other people might react negatively to my involvement”, and (12) “Not a priority for me”. To derive a scale of barriers to climate change action, a principal component analysis for categorical data assessed the factor structure of the 12 items. This analysis identified 1 factor consisting of the first 10 items, explaining 24.27% of the variance with an Eigenvalue of 2.9 and a Cronbach alpha of 0.73.

The response categories for race/ethnicity included “White”, “Black”, “Asian”, “Hispanic”, “Mixed”, or “Other”. Due to the small sample size, “Mixed” and “Other” were collapsed into a single category. Political ideology was assessed with the question “Where would you place yourself on a scale running from ‘Very liberal’ to ‘Very conservative’?” The response categories were (1) “Very liberal”, (2) “Liberal”, (3) “Slightly liberal”, (4) “Moderate”, (5) “Slightly conservative”, (6) “Conservative”, (7) “Very conservative”, and (8) “Not applicable”. Those who reported “Not applicable” were recoded to the median (n = 13). Political party affiliation was assessed with the question “Do you consider yourself Republican, Democrat, Independent, Libertarian, or Other?” Due to the small cell size, the Libertarian and the “Other” groups were collapsed into the “Other” group. Family income was dichotomized based on the median, at less than USD 60,000 versus USD 60,000 or more. Educational attainment was dichotomized at the median as a bachelor’s degree and higher versus an associate degree or less. Sex was assessed as biological sex assigned at birth as “female” and “male”.

### 2.2. Analysis

Of the 868 respondents who passed the validity check and completed the survey, 599 (69%) reported that climate change was “Extremely important” or “Very important” to them personally, and were included in the subsequent analysis. Bivariate and multivariate multinomial regression models examined the association between the independent variables and the three outcomes of recency of CCA communications (talking, posting on social media, and email/texting). The multinomial logistic regression models compared factors that predict the odds of engaging in the behavior in the prior month compared to never engaging in the behavior, and the odds of engaging in the behavior more than a month ago compared to never engaging in the behavior. For the multivariable models, the demographic variables (age, race, gender, education, and income) were entered into the models. Other predictors with a *p*-value of 0.15 or less in the bivariate analyses were included in the multivariable models. Due to the strong association between political party affiliation and political conservatism, the two variables were included in separate models. There were no pronounced differences in the models; hence, the models with political ideology, which allowed for treating political ideology as a continuous rather than a categorical variable, were presented. We examined multicollinearity with a correlation analysis of the variables. Then, we assessed VIF using a linear outcome and found no indication of multicollinearity. The multinomial analyses presented in the results focused on comparing CCA communications in the prior month to “never” rather than comparing CCA communications more than a month ago to “never” since recent behavioral reports are more likely to be accurate compared to reports on more distal events. In a post hoc analysis, we identified the barriers most strongly associated with CCA communications. Due to the large number of variables that assessed barriers and the anticipated correlation among the variables, we used a forward stepwise regression approach to identify the statistically significant (*p* < 0.05) variables for inclusion in the final models.

## 3. Results

There were 599 respondents included in the analysis who reported that the issue of global warming was “Extremely important” or “Very important” to them. The mean age was 33.4 (SD = 10.7). In terms of race, 40.1% were White, 33.9% non-Hispanic Black, 13.0% Hispanic, 5.5% Asian, and 7.5% were other. A majority (52.4%) of participants were born male, and 46.4% had an annual income of USD 60,000 or more. Most identified as liberal (73.3%), and 55.6% had a bachelor’s degree or higher (Table 1).

Of the respondents included in the analyses, 31.4% had talked to family/friends about CCA in the prior month, 45.1% talked to family/friends about CCA more than a month ago, and 23.5% had never talked about CCA. A total of 17.5% texted/emailed friends/family about CCA in the prior month, 33.7% texted/emailed friends/family about CCA more than a month ago, and 48.8% had never done so.

About half (49%, 92/188) of those who had talked about CCA to family and friends in the prior month had also emailed family and friends in the last month, and a similar number (48%) had posted on social media. Among those who posted in the prior month, 44.2% (62/140) had also emailed. Only 13.4% (55/411) who had not talked about CCA to family and friends in the prior month had in the prior month emailed/texted family or friends or posted on social media messages about climate change actions. Among the sample, 1.1% reported posting about climate change daily, 9.0% weekly, 10.7% less than weekly, and 35.9% less than monthly.

Although some demographic variables (age and sex) were not associated with talking, emailing/texting, or posting about CCA in the bivariate models, all of them were retained for the multivariate logistic regression models (Tables 2–4). Social norm variables were significant predictors of all three CCA communications modalities. Reporting more family/friends involved in climate change action significantly increased the odds of each of the three CCA communication modalities in the past month (talking aOR: 2.81, 95% CI: 1.93, 4.10; texting/emailing aOR: 2.16, 95% CI: 1.59, 2.95; posting on social media aOR: 2.00, 95% CI: 1.49, 2.69) and more than a month ago in bivariate and adjusted models. Similarly, reporting that family/friends would encourage climate change action significantly increased odds of talking (aOR: 1.59, 95% CI: 1.26, 2.00) and posting on social media about climate change action (aOR: 1.27, 95% CI: 1.03, 1.57) in the past month in bivariate and adjusted models, as well as more than a month ago. Reporting family/friends would encourage climate change action increased odds of past month texting/emailing about climate change action in the adjusted (aOR: 1.51, 95% CI: 1.20, 1.90) model but not in the bivariate model, but was a significant predictor of texting/emailing about climate change action more than a month ago in both models.

The climate distress score significantly increased the odds of CCA communicating across the three modalities in the past month (talking to family/friends aOR: 1.08, 95% CI: 1.01, 1.17; texting or emailing family/friends aOR: 1.15, 95% CI: 1.06, 1.24; posting or sharing posts on social media aOR: 1.15, 95% CI: 1.07, 1.23). Climate distress predicted talking to family/friends and texting or emailing family/friends more than a month ago.

An increased score on the measure of climate discomfort due to peers was only significantly associated with past month talking about CCA (aOR: 3.65, 95% CI: 1.99, 6.68) and more than a month ago. Increased youth actions was only associated with posting about CCA on social media in the past month (aOR: 2.03, 95% CI: 1.18, 3.50) and more than a month ago, and talking with family/friends more than a month ago.

Reporting a greater number of barriers to climate change action was significantly associated with lower odds of past month CCA communicating for all three modalities (talking to family/friends aOR: 0.78, 95% CI: 0.69, 0.87; texting or emailing family/friends aOR: 0.75, 95% CI: 0.66, 0.84; posting or sharing posts on social media aOR: 0.82, 95% CI: 0.74, 0.91), but in more than a month ago models, was only associated with talking with family/friends about CCA. Only a few demographic variables were associated with CCA communication. Non-Hispanic Blacks, compared to white respondents, had greater odds of past month posting or sharing about CCA (aOR: 2.07, 95% CI: 1.19, 3.61), and Hispanic, compared to white participants, were more likely to report texting/emailing more than a month ago.

In a post hoc analysis, the 10 individual items included in the scale of barriers to climate change activism were included using a forward stepwise multivariable multinomial regression model (Table 5). For the outcome of posted or shared a post about the need for action on climate change in the prior month compared to never, two items were statistically significant (“Activities like letter writing aren’t appealing to me”, and “Not something I think about a lot”.) in the prior month compared to never. For the outcome of talked to peers about climate change action in the prior month compared to never, three items were statistically significant: “Other people are better at it than I am”, “I’m not encouraged to become involved”, and “Not something I think about a lot”. The three barriers that differentiate the outcome of texted/emailed peers about climate change action in the prior month compared to never were “Other people are better at it than I am”, “Activities like letter writing aren’t appealing to me”, and “Not something I think about a lot”. As seen in Table 5, the odd ratios ranged from 0.23 to 0.47.

## 4. Discussion

The current study examined factors associated with CCA communications in a sample of US residents. In this study, a substantial number of adults who were concerned about climate change engaged in communications about climate change action. Conversations in the prior month with peers was the most frequent method of communication (31.4%), followed by posting on social media (23.4%). The third most frequent mode of communicating about CCA was email/texting (17.5%). Only a small proportion (13%) of respondents who had not talked about CCA to peers in the prior month had emailed peers or posted on social media about CCA in the prior month, which suggests that talking about climate change action was a primary CCA communication modality.

As documented in Tables 2–4, social norms were consistently associated with engaging in the three CCA communication modalities in the prior month, with the measure of descriptive norms having a stronger association than injunctive norms. Even though these two norms are correlated, the multivariable analyses suggested that they had independent associations with CCA communications. The role of social norms in promoting environmental behavior is well-documented [16,18,41], although an alternative explanation for these findings is a differential association with activists interacting with other activists. This positive association between both descriptive and injunctive social norms related to CCA communication is consistent with a more ritual view of communication, whereby, such communication is understood as a process that establishes shared meanings and beliefs that are in turn linked with perceptions of what one thinks one should do, as well as what one perceives others to do be doing.

These findings suggest that organizations that address climate change should encourage their members to communicate their climate change activities to peers. Changing norms by publicizing one’s behavior is a promising approach, since people who are already engaged in climate change actions are likely to realize that major efforts are needed to enact meaningful programs and policies for climate change mitigation and adaptation; hence, these activists may be open to publicizing their behaviors. Moreover, highlighting descriptive norms can model prosocial collective actions. Positive feelings and cognitions about engaging in these activities can also be modeled and shared on social media. To enhance the promotion of descriptive norms, future research should examine what information and images people who are engaged in climate change actions would be most interested in sharing, as well as cueing them to communicate their climate change actions.

Our findings suggest that people who recently communicated about CCA are also experiencing greater climate change distress than those who do not communicate about CCA (Tables 2–4). This distress should not be viewed as irrational or not well-founded. Climate change is a significant threat to species on earth and will result in massive morbidity and mortality. Distress can be viewed as a rational response to this threat. From a more ritual view of communication, CCA communication may establish mutual feelings and shared experiences that foster connection, build social relationships, and open up opportunities for the sharing of social support. Although we did not examine how climate change distress may be linked to psychopathology, it would be prudent to ensure that people who are involved in climate change activism behaviors do not experience overwhelming distress that significantly impedes their quality of life. Climate change organizations that foster CCA communication should provide recommendations on stress management, which may include social support and forums to discuss well-being, as well as training in evidence-based methods to address distress, such as mindfulness, meditation, exercise, and activities in nature. To enhance resilience, organizations that address climate change can build on this dialogical understanding of CCA to build opportunities to provide social support, through appraisal or emotional support, to their members that highlight the meaningfulness of their actions and reinforce the shared meanings and beliefs held by their community of members [42,43].

Barriers to climate change action, as assessed by the scale, were associated with using each of the three CCA communication modalities in the prior month (Tables 2–4). The principal component analysis of barriers to engaging in climate change action revealed that 10 out of 12 items loaded on one factor. This finding suggests that many barriers are correlated, and that there may be a latent factor influencing barriers to CCA. Future research should explore both quantitatively and qualitatively the intersections of reported barriers to CCA to examine the underlying factors that influence the barriers people report. Furthermore, longitudinal studies are needed to assess the relationship between barriers to climate change actions and CCA communications over time. In examining individual barriers to CCA communications, the barrier of “not something I think a lot about” was strongly associated with the three CCA communication modalities in the prior month. This finding suggests that it is critical to increase communications addressing the perceived severity of climate change, as well as individuals’ perceived susceptibility to the effects of climate change. Future research should examine how to link people’s other priorities with climate change actions so that it becomes a higher priority in their lives. A complementary approach is to reduce barriers to engaging in climate change actions and cue behaviors. However, actions such as making contact with policymakers easier by having respondents send a form letter to their legislators may indicate that constituents are not highly invested in the topic, and reminders from climate change organizations may appear to be solicitations for donations [44].

Not being encouraged to become involved in climate change activism was negatively associated with talking to family and friends. This barrier suggests that it is important to remind people of the importance of engaging in climate change actions, especially when they have competing priorities, and cue behaviors to engage in climate change action. Careful attention to who provides these reminders is needed, with the trust and influence of the source of the messages being critical to evaluate. Different audiences may likely benefit from distinct messengers. The reported barrier of “activities like letter writing not being appealing” was significantly associated with posting on social media and texting/emailing friends/family about climate change action. These associations suggest the importance of training people in climate change action as well as highlighting how activities such as letter writing can be made more engaging. Approaches such as asking people why addressing climate change is important to them may increase the appeal of written communication. Providing exemplar posts and texts that people may want to share may also reduce communication barriers.

One substantial difference between modes of prior month CCA communication was that endorsing the statement “I would feel very uncomfortable telling my friends/family if I was involved in actions to combat climate change” was only associated with talking to friends/family about climate change, and not email/texting or posting on social media. This finding suggests that for a subgroup (18% in the current sample), it may be more effective to ask them to post or email/text rather than talk face-to-face. Activities deemed political may be viewed as incurring negative repercussions among some of their peers. Prior research suggests that political and especially environmental activists are viewed negatively by some sectors of the population [42,45]. Future research should examine how to frame climate change activism so that it has a positive valence for those who do not want to engage in political activities. Future research can also examine where youth climate change actions are publicized and observed. It may be that these actions tend to be seen more on social media than other news media, which may help to explain this association [46].

There were few demographics correlated with recency of CCA communications, with the exception that Black participants, compared to white participants, reported greater odds of posting in the prior month; Hispanic participants, compared to white participants, reported greater odds of texting/emailing more than a month ago compared to never. It was surprising, however, that political conservatism and political party affiliation were not associated with any of the three CCA communication behaviors. Prior studies have found lower levels of concern about climate change among Republicans compared to Democrats [47]. One potential explanation for this finding is that this study only included individuals who reported that climate change was a very or extremely important topic to them. Although few demographic differences were detected in this sample, we do not know if the same messages and trainings to promote climate change action will be invariant based on demographic characteristics, and future research should examine in more detail demographic differences in training needs and interests and message appeal. It is also important to determine what messages and messengers will be most effective. An analysis of Greta Thunberg’s Instagram posts suggests that she frames the topic of climate change as an ethical issue, employs an emotional appeal of hope, and visually frames collective action [48]. Future research should also examine how the format and content of messages may influence climate change actions, as some prior studies have not found that experimental manipulations of messages have a significant impact on behaviors [49–51]. In addition to focusing on motivating people to become involved in CCA communications, it is also important to ensure that their CCA communications are sustained.

Study limitations should be noted. This was not a random sample, and hence the study results cannot be generalized to the US population. However, there is strong evidence of the validity of Prolific respondents’ data compared to other platforms and national representative panels [38]. The cross-sectional study design reduces causal inferences. For example, descriptive and injunctive social norms related to CCA may influence CCA communication, but the inverse may also be true as well. Future research should investigate these relationships longitudinally to explore the ways in which CCA communication influences, and is influenced by, community norms to identify opportunities for collective action. In addition, the self-reports of CCA communications were not verified by external measures. There may have been unmeasured barriers, and the measures of social norms and social influences were limited. We also did not measure in detail the frequency of communications, which could help identify individuals who are most highly active in CCA communications.

Findings from this study suggest that social norms are strongly linked to CCA communications, as are climate change distress and barriers to climate change action. Discomfort in telling friends/family about being involved in collective actions to combat climate change was associated with recency of talking about climate change action. These findings suggest the importance of social influence on CCA communications. This dynamic should be taken into account when developing programs for climate change collective actions. Given the necessity of organizing large numbers of people to combat climate change, it is critical to utilize social norms and other forms of social influence to enact policies to address climate change.

## Figures and Tables

**Table 1.** Demographic data among respondents who report that the issue of climate change is extremely or very important to them (N = 599).

Variable	*n* (%)
Age, mean (SD)	33.35 (10.73)
Race	
White	240 (40.07)
Non-Hispanic Black	203 (33.89)
Hispanic	78 (13.02)
Asian	33 (5.51)
Other	45 (7.51)
Sex	
Male	314 (52.42)
Female	285 (47.58)
Income	
Less than USD 60K	321 (53.59)
Greater than USD 60K	278 (46.41)
Education	
Associate degree or less	266 (44.41)
Bachelor’s degree or higher	333 (55.59)
Political Ideology	
Very Liberal	171 (28.55)
Liberal	178 (29.72)
Slightly Liberal	90 (15.03)
Moderate	98 (16.36)
Slightly Conservative	23 (3.84)
Conservative	28 (4.67)
Very Conservative	11 (1.84)

(%) unless otherwise noted, SD = standard deviation.

**Table 2.** Bivariate and multivariable multinomial logistic regression models of correlates of talking to family and friends about climate change action among respondents who report that the issue of climate change is very or extremely important to them.

	Bivariate Multinomial Logistic Regressions (OR, 95% CI) (Ref: Never Talked about Climate Change Action)		Multivariable Multinomial Logistic Regression (aOR, 95% CI) (Ref: Never Talked about Climate Change Action)	
	Talked about Climate Change Action More Than a Month Ago	Talked about Climate Change Action in the Past Month	Talked about Climate Change Action More Than a Month Ago	Talked about Climate Change Action in the Past Month
Age (years)	1.00 (0.98, 1.02)	1.01 (0.99, 1.03)	1.01 (0.99, 1.03)	1.02 (0.99, 1.04)
Sex (Ref: Male)	0.74 (0.49, 1.11)	0.76 (0.49, 1.17)	0.62 (0.39, 1.00)	0.75 (0.44, 1.28)
Race (Ref: White)	REF	REF	REF	REF
Non-Hispanic Black	0.92 (0.57, 1.48)	0.91 (0.55, 1.51)	0.89 (0.52, 1.53)	0.89 (0.49, 1.65)
Hispanic	0.96 (0.50, 1.82)	0.83 (0.41, 1.67)	1.16 (0.55, 2.43)	1.25 (0.53, 2.91)
Asian	0.81 (0.34, 1.90)	0.48 (0.17, 1.33)	0.66 (0.25, 1.75)	0.49 (0.15, 1.61)
Other	1.23 (0.53, 2.86)	1.06 (0.43, 2.63)	1.61 (0.64, 4.05)	1.73 (0.61, 4.90)
Education (Ref: Associate degree or less)	**1.77 (1.17, 2.66)**	**2.38 (1.52, 3.73)**	1.36 (0.84, 2.20)	1.47 (0.85, 2.55)
Income (Ref: USD 60K or less)	1.48 (0.97, 2.24)	**1.99 (1.27, 3.10)**	1.13 (0.69, 1.83)	1.29 (0.75, 2.24)
Political orientation (liberal to conservative)	**0.86 (0.75, 0.98)**	0.91 (0.79, 1.05)	0.92 (0.77, 1.09)	0.96 (0.79, 1.17)
Climate change distress	**1.06 (1.01, 1.11)**	1.03 (0.97, 1.08)	**1.07 (1.00, 1.15)**	**1.08 (1.01, 1.17)**
Discomfort due to anticipated peer reaction	**1.76 (1.14, 2.71)**	**3.24 (1.94, 5.41)**	**1.82 (1.12, 2.95)**	**3.65 (1.99, 6.68)**
Youth action	**2.22 (1.44, 3.42)**	**2.16 (1.36, 3.45)**	**1.66 (1.02, 2.72)**	1.35 (0.77, 2.37)
Barriers to climate change action	**0.87 (0.80, 0.95)**	**0.75 (0.68, 0.82)**	**0.88 (0.80, 0.97)**	**0.78 (0.69, 0.87)**
Family/friends involved in climate change action	**2.71 (1.96, 3.73)**	**3.96 (2.82, 5.55)**	**2.20 (1.54, 3.12)**	**2.81 (1.93, 4.10)**
Family/friends would encourage climate change action	**1.64 (1.35, 1.98)**	**2.17 (1.76, 2.66)**	**1.28 (1.04, 1.58)**	**1.59 (1.26, 2.00)**

Notes: OR—odds ratio, CI—confidence interval, aOR—adjusted odds ratio, Ref: reference group, Bold: *p* < 0.05.

**Table 3.** Bivariate and multivariable multinomial logistic regression models of correlates of texting and emailing family and friends about climate change action among respondents who report that the issue of climate change is very or extremely important to them.

	Bivariate Multinomial Logistic Regressions (OR, 95% CI) (Ref: Never Texted/Emailed about Climate Change Action)		Multivariable Multinomial Logistic Regression (aOR, 95% CI) (Ref: Never Texted/Emailed about Climate Change Action)	
	Texted/Emailed about Climate Change Action More Than a Month Ago	Texted/Emailed about Climate Change Action in the Past Month	Texted/Emailed about Climate Change Action More Than a Month Ago	Texted/Emailed about Climate Change Action in the Past Month
Age (years)	0.99 (0.98, 1.01)	1.00 (0.98, 1.02)	1.00 (0.98, 1.02)	1.00 (0.97, 1.02)
Sex (Ref: Male)	0.97 (0.68, 1.39)	0.65 (0.41, 1.02)	1.00 (0.67, 1.49)	0.76 (0.44, 1.29)
Race (Ref: White)	REF	REF	REF	REF
Non-Hispanic Black	0.94 (0.62, 1.45)	1.07 (0.64, 1.77)	0.97 (0.60, 1.54)	1.02 (0.56, 1.85)
Hispanic	**1.90 (1.09, 3.32)**	0.91 (0.41, 2.01)	**2.29 (1.24, 4.22)**	1.26 (0.52, 3.05)
Asian	1.82 (0.84, 3.94)	0.60 (0.17, 2.20)	1.72 (0.74, 3.98)	0.82 (0.20, 3.41)
Other	0.83 (0.39, 1.77)	1.22 (0.54, 2.78)	0.96 (0.43, 2.15)	1.58 (0.62, 4.02)
Education (Ref: Associate degree or less)	**1.71 (1.19, 2.47)**	**2.30 (1.44, 3.67)**	1.41 (0.93, 2.13)	1.42 (0.81, 2.49)
Income (Ref: USD 60K or less)	**1.62 (1.13, 2.33)**	**1.71 (1.09, 2.68)**	1.20 (0.80, 1.80)	0.98 (0.57, 1.70)
Political orientation (liberal to conservative)	0.93 (0.82, 1.05)	1.07 (0.93, 1.23)	1.01 (0.87, 1.17)	1.17 (0.97, 1.40)
Climate change distress	**1.05 (1.00, 1.09)**	1.03 (0.98, 1.09)	**1.06 (1.00, 1.12)**	**1.15 (1.06, 1.24)**
Discomfort due to anticipated peer reaction	1.23 (0.82, 1.85)	1.36 (0.81, 2.30)	1.18 (0.76, 1.84)	1.41 (0.76, 2.60)
Youth action	**1.86 (1.24, 2.77)**	**1.95 (1.17, 3.26)**	1.35 (0.87, 2.09)	1.21 (0.67, 2.19)
Barriers to climate change action	**0.93 (0.86, 1.00)**	**0.73 (0.66, 0.81)**	0.93 (0.85, 1.01)	**0.75 (0.66, 0.84)**
Family/friends involved in climate change action	**1.99 (1.58, 2.52)**	**3.03 (2.31, 3.97)**	**1.79 (1.37, 2.33)**	**2.16 (1.59, 2.95)**
Family/friends would encourage climate change action	**1.35 (1.16, 1.58)**	**1.89 (1.55, 2.29)**	1.14 (0.96, 1.36)	**1.51 (1.20, 1.90)**

Notes: OR—odds ratio, CI—confidence interval, aOR—adjusted odds ratio, Ref: reference group, Bold: *p* < 0.05.

**Table 4.** Bivariate and multivariable logistic regression models of correlates posting or sharing a post on social media about the need for climate change action among respondents who report that the issue of climate change is very or extremely important to them.

	Bivariate Multinomial Logistic Regressions (OR, 95% CI) (Ref: Never Posted or Shared Climate Change Action Posts)		Multivariable Multinomial Logistic Regression (aOR, 95% CI) (Ref: Never Posted or Shared Climate Change Action Posts)	
	Posted/Shared a Post about Climate Change Action More Than a Month Ago	Posted/Shared a Post about Climate Change Action in the Past Month	Posted/Shared a Post about Climate Change Action More Than a Month Ago	Posted/Shared a Post about Climate Change Action in the Past Month
Age (years)	1.00 (0.98, 1.02)	1.01 (0.99, 1.03)	1.00 (0.98, 1.02)	1.02 (0.99, 1.04)
Sex (Ref: Male)	1.15 (0.80, 1.66)	1.05 (0.69, 1.59)	1.23 (0.82, 1.85)	1.16 (0.71, 1.90)
Race (Ref: White)	REF	REF	REF	REF
Non-Hispanic Black	1.06 (0.69, 1.64)	**1.68 (1.04, 2.70)**	1.05 (0.65, 1.69)	**2.07 (1.19, 3.61)**
Hispanic	1.51 (0.85, 2.67)	1.00 (0.48, 2.06)	1.53 (0.82, 2.87)	1.22 (0.54, 2.75)
Asian	0.65 (0.29, 1.49)	0.56 (0.20, 1.58)	0.48 (0.20, 1.18)	0.56 (0.17, 1.81)
Other	1.72 (0.84, 3.52)	1.07 (0.42, 2.68)	2.13 (0.98, 4.62)	1.48 (0.54, 4.08)
Education (Ref: Associate degree or less)	**1.70 (1.18, 2.47)**	**1.70 (1.11, 2.59)**	1.42 (0.93, 2.18)	0.99 (0.59, 1.65)
Income (Ref: USD 60K or less)	**1.85 (1.28, 2.69)**	**1.76 (1.15, 2.69)**	1.48 (0.97, 2.26)	1.40 (0.85, 2.33)
Political orientation (liberal to conservative)	**0.86 (0.76, 0.97)**	0.93 (0.81, 1.06)	0.88 (0.76, 1.03)	1.00 (0.84, 1.19)
Climate change distress	1.02 (0.98, 1.07)	**1.06 (1.01, 1.12)**	0.98 (0.93, 1.04)	1.15 (1.07, 1.23)
Discomfort due to anticipated peer reaction	1.14 (0.75, 1.72)	1.23 (0.76, 1.98)	1.08 (0.69, 1.70)	1.15 (0.66, 1.99)
Youth action	**2.49 (1.66, 3.73)**	**3.11 (1.90, 5.09)**	**2.05 (1.32, 3.18)**	**2.03 (1.18, 3.50)**
Barriers to climate change action	0.99 (0.92, 1.06)	**0.80 (0.74, 0.88)**	1.03 (0.95, 1.12)	**0.82 (0.74, 0.91)**
Family/friends involved in climate change action	**1.63 (1.29, 2.06)**	**2.51 (1.95, 3.25)**	**1.36 (1.04, 1.78)**	**2.00 (1.49, 2.69)**
Family/friends would encourage climate change action	**1.48 (1.27, 1.74)**	**1.67 (1.40, 2.00)**	**1.29 (1.09, 1.54)**	**1.27 (1.03, 1.57)**

Notes: OR—odds ratio, CI—confidence interval, aOR—adjusted odds ratio, Ref: reference group, Bold: *p* < 0.05.

**Table 5.** Multivariable forward stepwise logistic regression models of climate change communication actions adjusting for reported barriers to climate action included on the barriers to climate action scale. ˆ (N = 599).

Climate Change Communication Actions in the Prior Month Compared to Never Engaging in the Actions	Self-Reported Barriers	Wald	aOR (95% CI)
Posted or shared a post about the need for action on climate change	Activities like letter writing aren’t appealing to me	15.858	0.40 (0.25–0.62)
	Not something I think about a lot *	16.141	0.23 (0.11–0.47)
Talked to peers about climate change action	Other people are better at it than I am	12.574	0.43 (0.27–0.69)
	I’m not encouraged to become involved	8.660	0.44 (0.25–0.76)
	Not something I think about a lot *	7.276	0.45 (0.25–0.80)
Texted/emailed peers about climate change action	Other people are better at it than I am	13.557	0.37 (0.22–0.63)
	Activities like letter writing aren’t appealing to me *	8.842	0.47 (0.29–0.77)
	Not something I think about a lot *	11.064	0.25 (0.12–0.56)

* Statistically significant when comparing “more than a month ago” to “never”.

ˆ using forward entry into the stepwise regression models for the three climate communications actions as dependent variables with a criterion of *p* < 0.05 to enter into the models. Ten barriers were tested for inclusion in each of the three models. aOR—adjusted odds ratio, CI—confidence interval.

## Data Availability

The datasets generated during and analyzed during the current study are not publicly available due to the ongoing study, but are available from the corresponding author upon reasonable request.
